# Bangla Sign Language (BdSL) Alphabets and Numerals Classification Using a Deep Learning Model

**DOI:** 10.3390/s22020574

**Published:** 2022-01-12

**Authors:** Kanchon Kanti Podder, Muhammad E. H. Chowdhury, Anas M. Tahir, Zaid Bin Mahbub, Amith Khandakar, Md Shafayet Hossain, Muhammad Abdul Kadir

**Affiliations:** 1 Department of Biomedical Physics & Technology, University of Dhaka, Dhaka 1000, Bangladesh; kanchon.k.podder@bmpt.du.ac.bd (K.K.P.); kadir@du.ac.bd (M.A.K.); 2 Department of Electrical Engineering, Qatar University, Doha 2713, Qatar; a.tahir@qu.edu.qa (A.M.T.); amitk@qu.edu.qa (A.K.); 3 Department of Mathematics and Physics, North South University, Dhaka 1229, Bangladesh; zaid.mahbub@northsouth.edu; 4 Department of Electrical, Electronic and Systems Engineering, Universiti Kebangsaan Malaysia, Bangi 43600, Selangor, Malaysia; p108100@siswa.ukm.edu.my

**Keywords:** bangla sign language, alphabets and numerals, classification, convolutional neural network, semantic segmentation

## Abstract

A real-time Bangla Sign Language interpreter can enable more than 200 k hearing and speech-impaired people to the mainstream workforce in Bangladesh. Bangla Sign Language (BdSL) recognition and detection is a challenging topic in computer vision and deep learning research because sign language recognition accuracy may vary on the skin tone, hand orientation, and background. This research has used deep machine learning models for accurate and reliable BdSL Alphabets and Numerals using two well-suited and robust datasets. The dataset prepared in this study comprises of the largest image database for BdSL Alphabets and Numerals in order to reduce inter-class similarity while dealing with diverse image data, which comprises various backgrounds and skin tones. The papers compared classification with and without background images to determine the best working model for BdSL Alphabets and Numerals interpretation. The CNN model trained with the images that had a background was found to be more effective than without background. The hand detection portion in the segmentation approach must be more accurate in the hand detection process to boost the overall accuracy in the sign recognition. It was found that ResNet18 performed best with 99.99% accuracy, precision, F1 score, sensitivity, and 100% specificity, which outperforms the works in the literature for BdSL Alphabets and Numerals recognition. This dataset is made publicly available for researchers to support and encourage further research on Bangla Sign Language Interpretation so that the hearing and speech-impaired individuals can benefit from this research.

## 1. Introduction

The global population is made up of 15% of the population who has various forms of disabilities. There are over five percent of the population that is deaf, which is over 466 million people. According to the World Health Organization (WHO), the population that may be expanded to 500 million by 2050 is about 2.7 times more than the population of the year 2000. At least 70 million individuals have their speech and hearing capabilities affected.

These people deal with difficulties in interacting with others especially when joining the workforce, education, healthcare, and transportation. In a survey conducted in the United States (US) that explored healthcare access for deaf women, the study discovered that the healthcare service providers neglected to teach them how to interact with other individuals [1]. Conversely, the United Nations Convention on the Rights of Persons with Disabilities (UNCRPD) guarantees the use of sign language and supports deaf and the sign language by safeguarding these populations [2]. People who have hearing and speech disabilities also need interpreters to communicate with the hearing and speech-capable population [3]. However, assigning and training interpreters in underprivileged and remote areas is difficult [4,5]. Thus, those groups of individuals are missing out on a vital necessity for all human beings to have a normal life like others in underdeveloped nations, developing nations, and affluent nations alike [6].

According to Department of Social Services et al. [7], there are 153,776 vocal disable people, 73,507 hearing disable people, and 9625 hearing and visually disabled people in Bangladesh. The popular and, in most cases, the only medium of communication of hearing and speech-disabled people is sign language. However, this medium of communication is not effective when speech and hearing disabled people communicate with people who do not know sign language. A digital Bangla Sign Language Interpretation system can surpass this communication barrier between vocal-hearing disable people and a common person.

In this research, a system is built for real-time Bangla Sign Alphabets Numerals interpretation to minimize the barrier between a sign language user and a non-sign language user. The main contributions of this research are as follows:

- A large Bangla Sign Alphabets and Numerals dataset was developed for both one-handed and two-handed representation.
- A Bangla Sign Alphabets and Numerals recognition system using transfer learning on three different pre-trained deep convolutional neural networks (CNNs) was proposed.
- Hand detection using semantic segmentation and then recognition of Bangla Sign Alphabets and Numerals using transfer learning on the same three pre-trained CNNs was also proposed.
- The best model in this study exceeded previous state-of-the-art efforts in the recognition of Bangla Sign Alphabets and Numerals.
- Developed a real-time Bangla Sign Alphabets and Numerals interpreter.

The rest of the paper is organized as such: Section 1 gives a brief introduction to the research. Section 2 demonstrates the literature review. Section 3 provides the methodology of research comprising dataset description, proposed pipeline with approaches, algorithm, and details of the experiments. Section 4 presents the findings of the investigations, followed by a conclusion in Section 5, and, lastly, the recommendations are presented in Section 6.

## 2. Literature Review

One-hand and two-hand are the two methods to represent Bangla Sign Alphabets. Both of the representation systems have been used in Bangla Sign Language Recognition research over the years. A computer vision-based two hands BdS Alphabets recognition system developed in Deb et al. [8] used normalized cross-correlation. Using a neural network ensemble, Ref. [9] achieved 93% accuracy in BdSL recognition. In Uddin et al. [10], few BdS alphabets were recognized with the application of image processing, vector quantization, and support vector machine (SVM). Sensitivity towards background and illuminations are the two most concerning factors in sign language recognition. Refs. [11,12,13] discussed these two issues and proposed a computer vision-based solution in Bangla Sign Language Recognition. Application of OpenNI framework and Artificial Neural Network on images captured using Kinect for recognition of few Bangla Sign words was proposed in Choudhury et al. [14]. In Jarman et al. [15], a fingertip finding algorithm was used for BdSL recognition. CNN is also popular in recognition of BdSL [16]. VGG19 CNN was used in Rafi et al. [17] for recognition of one hand BdS alphabets and achieved 89.6% accuracy. Only 15 different gestures were reported to be recognized by the proposed system in [18]. Color-coded fingertips and ResNet18 were used in Podder et al. [19] for recognition of 37 Bangla Sign Alphabets.

Deep learning is leveraging the field of computer vision in different aspects such as autonomous driving [20], biomedical applications [21,22,23], etc., to name a few. Segmentation and visualization techniques are often used in machine learning technique to confirm the reliability of the trained model and, in fact, segmentation has helped in improving the classification performance [24,25,26]. To increase the reliability of the classification models, semantic segmentation models are used in sign language [27]. Visualization techniques [28,29,30] are also another method used in different tasks to understand whether the model is trained on useful features or not when performing classification or recognition task [26]. Thus, deep learning techniques along with different visualization techniques were adopted to this research for Bangla Sign Language Alphabets and Numerals recognition.

## 3. Methods and Materials

**Figure 1:**
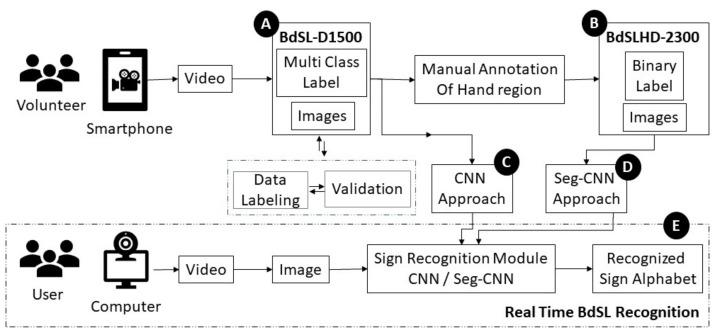
Overview of the method and materials.

Bangla Sign Language recognition and detection is a challenging topic in computer vision and deep learning research because sign language recognition accuracy may vary on the skin tone, hand orientation, and background. Counting all these challenges, this research has been done in two approaches for the investigation of the Bangla Sign Language Interpretation with two well-suited datasets. Figure 1 represents the overview of methods and materials applied in this research.

### 3.1. Dataset Properties

For this research, the dataset has been collected from 20 volunteers from different backgrounds using a smartphone camera. Images were extracted from the videos taken by volunteers to create the dataset. A written informed consent for publication was obtained from participating subjects who can be identified.

#### 3.1.1. BdSL-D1500

**Figure 2:**
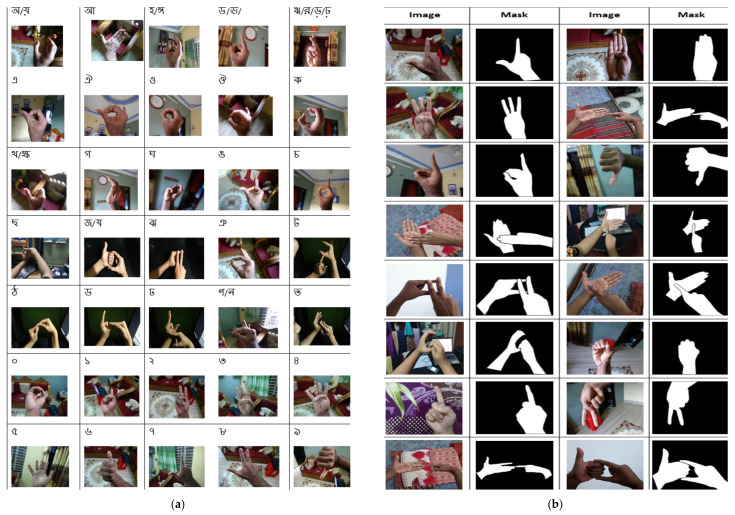
(**a**) Representation of the BdSL-D1500 Dataset, and (**b**) representation of BdSL-D1500 after applying the best trained model on BdSLHD-2300.

The Bangla Sign Language Dataset (BdSL-D1500) [31] (Figure 1 block (A)) which was collected for this research contains 87 classes of images which includes 38 gestures of one hand representation of BdS Alphabet, 36 gestures for two-hand representation, 10 BdS digits (0 to 9), two numerals (00, 000), and one gesture called “Counting” (গণনা). Figure 2a represents a sample of the overall BdSL-D1500 dataset.

- Each Class has approximately 1500 different images extracted from videos of different volunteers.
- In all classes, the background is different for different images.
- The total number of images in the dataset is approximately 132,061.
- Images that were extracted from videos are color images (RGB).

#### 3.1.2. BdSLHD-2300

Another dataset was also created for hand detection (Figure 1 block (B)). This hand detection dataset (BdSLHD-2300) was used to train the hand segmentation models. The properties of this dataset are given below:

- From each class of the Bangla Sign Language Dataset, around 27 images have been collected from BdSL-D1500 for BdSLHD-2300.
- The dataset contains approximately 87×27=2300 images.
- The hand in the image was annotated manually using MATLAB 2020 and created masks for 2300 images. The masks contained binary details, as the area of the hand was filled in white, while the other portion was considered as a background and filled in black.
- The dataset has both RGB images and the binary mask of the images (Figure 2b).

### 3.2. Data Validation and Preprocessing

The collected video from different volunteers was verified and validated to create the appropriate image dataset. As the videos were collected through crowdsourcing, unwanted and noisy videos or a portion of the videos were removed. All images in BdSL-D1500 and BdSLHD-2300 were resized to 331×331 resolution. The mean and standard deviation values were calculated for both of the datasets. In Table 1, the mean and standard deviation values for both BdSL-D1500 and BdSLHD-2300 are given:

### 3.3. Proposed Pipeline

Two approaches, such as classification with background and classification without background approaches, were used for Bangla Sign Alphabets and Numerals interpretation. For the training purpose, transfer learning was used for training the pre-trained Convolutional Neural Network (CNN) models. The layers of CNNs were not frozen and trained based on the weights of ImageNet classification [32,33].

#### 3.3.1. Classification with Background

**Figure 3:**
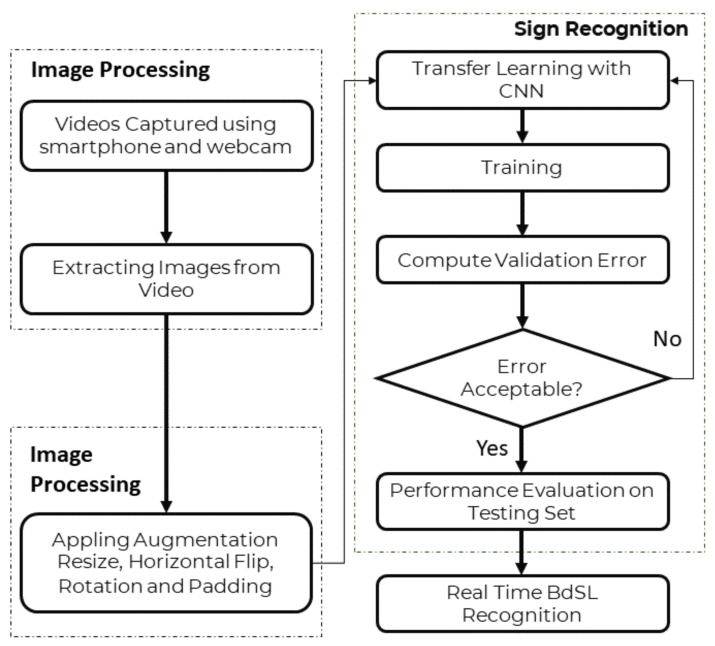
Flow diagram of BdSL interpretation in a classification with background approach.

Three pre-trained CNN models were used as the first approach to investigate the interpretation of Bangla Sign Alphabet on the Bangla Sign Language Dataset [31]. To avoid overfitting during training, online image augmentation techniques such as image resize, image rotation, horizontal flip, and image padding were used. A flow diagram of CNN based approach from the BdSL-D1500 dataset training to real-time BdSL interpretation is shown in Figure 3.

#### 3.3.2. Classification without Background Approach

**Figure 4:**
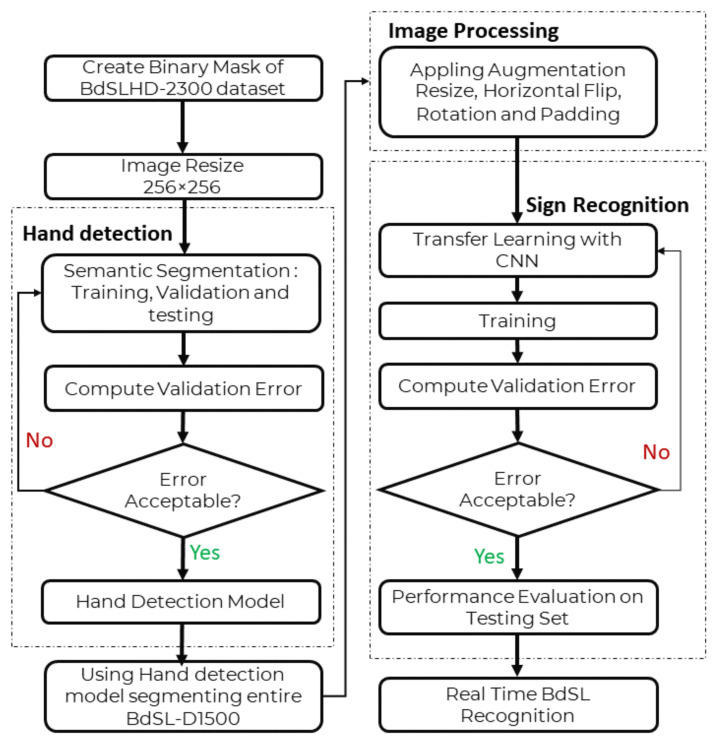
Flow diagram of BdSL interpretation in the classification without background approach.

As sign recognition using deep learning has susceptibility towards the background, the second approach of classification was performed in this study where the hand segmented images were used for training and testing. Firstly, the BdSLHD-2300 [34] dataset, which is a subset of the BdSL-D1500 dataset (1.74%), was created by manually editing the hand mask from the original images of the BdSLHD-2300 dataset (2.3 k). A hand detection model was developed by training several hand segmentation models, and the best segmentation model was identified. Using the best model, the BdSL-D1500 dataset (132 k) has been segmented. Figure 2b represents a sample of the BdSLHD-2300 dataset used for training the segmentation network. The newly background removed dataset is then trained on the same three CNN models for BdS Alphabets and Numerals recognition and interpretation. Figure 4 represents the entire work flow of the BdSl interpretation in the classification without background approach.

### 3.4. Classification and Segmentation Models

For classification, three pre-trained CNN models were used ResNet18 [35], MobileNet_V2 [36], and EfficientNet_B1 [37] and for semantic segmentation of hand region and background removal, three CNN models such as DenseNet201 Feature Pyramid Networks (FPN) [38], U-Net [39], and M-UNet [40] were used.

ResNet18 [35] is a deep residual learning framework, which is popular for its shortcut connections. Using this technique, Ref. [35] provided evidence of vanishing gradients and decreasing accuracy after saturation. MobileNet_V2 [36] was designed to replace expensive convolution networks with a cheaper network. Ref. [36] implemented expansion/projection layers and residual connections to make this network usable in mobile devices. It is also mentioned that removing nonlinearities in narrow layers is important for maintaining representational power. On the other hand, EfficientNet [37] is a new state-of-the-art CNN. The seed of the EfficientNet CNN family is Mobile Inverted Bottleneck Convolution (MBConv). The main working method of this CNN is to determine the appropriate scaling coefficient under a fixed resourced constraint by firstly doing a grid search on the relation among baseline networks’ distinct scaling dimensions.

Semantic segmentation is a technique to classify pixels in an image to corresponding labels. In a fully connected CNN models, the last layer can be replaced with convolution layers for semantic segmentation, but the feature map at the last layer is down-sampled by previous convolutional operations. For that, semantic segmentation networks have two parts: down-sampling and up-sampling parts to match the input image size with proper deconvolution in up-sampling. UNet, a convolutional network, has two parts. In the encoder part of UNet, the context of the picture is captured and, in the decoder part, the localization of the object is done. MUNet is a multi-scale U-Net framework, which has the same encoder–decoder as U-Net and connected with a skip connection. In a completely convolutional manner, FPN takes as input a single-scale picture of any size and produces as output proportionally scaled feature maps at numerous layers, all of which are proportionately sized. The main features of DenseNet201 FPN are reducing the number of parameters, reusing features, alleviating the vanishing gradient problem, and results in stronger feature propagation.

Different types of loss functions (Balanced Cross-Entropy, Dice Loss, and Negative Log-Likelihood) were used to investigate the performance of semantic segmentation of hand or hand detection models.

If *y* is true value and $\hat{y}$ is predicted value, the Balanced Cross Entropy loss function is given in Equation (1):

(1) $$L_{BCE}=-\left(B\times ylog\hat{y}+\left(1-B\right)\times \left(1-y\right)log\left(1-\hat{y}\right)\right)$$

where B=$\left(1-\frac{y}{H\times W}\right)$

If *y* is the binary label and $\hat{p}$ is the predicted probabilities, Equation (3) represents the Dice Loss,

(2) $$\mathrm{DL}\left(y,\hat{p}\right)=1-\frac{2y\hat{p}+1}{y+\hat{p}+1}$$

Negative log-likelihood (NLL) loss creates a penalty for model making correct prediction with lower probabilities. In multi-class classification, the logarithmic of NLL gives this penalty, and NLL is responsible for correct prediction with greater probabilities. The NLL loss expressed as

(3) NLL−Loss(x,y)=−(logy)

Here, *x* indicates the actual value, while *y* indicates the predicted value.

### 3.5. Visualization Technique

**Figure 5:**
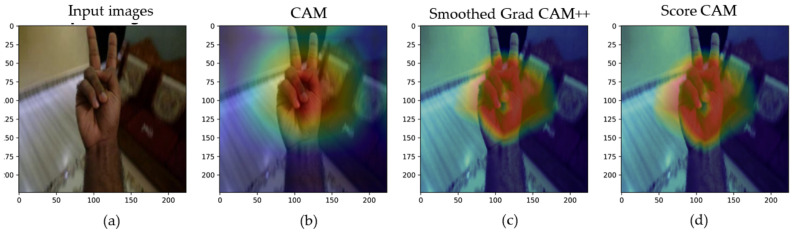
Input Images (**a**), CAM (**b**), Smoothed Grad-CAM++ (**c**), and Score-CAM visualization (**d**) of Bangla Sign Alphabet by a state-of-the-art CNN.

For understanding the reasoning underlying CNN prediction, there are a variety of methodologies available, including Class Activation Mapping (CAM) [28], Grad-CAM++ [29], Smoothed Grad-CAM++ [30], and Score-CAM [28]. The visualization techniques help users to put trust on the CNN by understanding the learned features by CNN. CAM needs global pooling layers [29] to track the desired convolutional layer and, for this reason, CAM is model sensitive [41] as not all models require a global pooling layer. Removing the model sensitiveness, smoothed Grad-CAM++ is a mixture of Smoothed GRAD and Grad-CAM++ that is capable of displaying several things throughout the model prediction process, such as a subset of feature maps, a convolutional layer, or a subset of neuron in a feature map [42]. Later, Ref. [28] introduced Score-CAM, in which the significance of activation maps is encoded. The encoding is based on the term of the global contribution of the associated input features rather than the local sensitivity measurements. Figure 5 represents a sample Bangla Sign alphabet visualization by CAM, Smoothed Grad-CAM++, and Score-CAM with the heat map overlying on the input image showing the hand region adopted by CNN in sign alphabet prediction. The Smoothed Grad CAM++ and Score-CAM address features learned by the model more accurately than CAM. In Figure 5, Smoothed Grad CAM++ and Score-CAM localized more region required hand shapes than the CAM localization. A detailed comparison and analysis are conducted in the Section 4.4. This may assist users in comprehending how the network makes choices. This may also help to increase end-user confidence if it can be established which portion of hand region for predicting Bangla Sign Alphabets and Numerals the network focuses on.

### 3.6. Experimental Setup

For Classification With or Without Background approach, a five-fold cross-validation scheme on BdSL-D1500 before and after segmentation and BdSLHD-2300 datasets for segmentation was used with a ratio of 70% training, 10% validation, and 20% testing. In this research, Google Colab Pro was used for training, validation, and testing with a 16 GB GPU facility of 12 GB RAM and 16 GB GPU (Tesla T4).

In hand detection using DenseNet201-FPN, UNet, and M-Unet, Stochastic Gradient Descent (SGD) was used as an optimizer with an initial learning rate of 0.001 and batch size of 16. Three different loss functions were investigated to evaluate the performance of the hand detection models. For sign recognition in CNN (classification with background), and in the second part of the classification without background approach SGD was used as optimizer for ResNet18, MobileNet_V2, while an Adam optimizer was used for EfficientNet_B1. Table 2 represents the training parameter used in hand detection and sign recognition.

### 3.7. Evaluation Metrics

For a hand detection segment and classification problem (with or without background), different parameters were used for quantitative analysis. The evaluation in hand detection is done on pixel-level analysis, where the background was counted as a negative class, and the hand region was counted as a positive class. The performance of the hand detection and sign recognition was done using several evaluation metrics with 90% confidence intervals(CI). Thus, the CI for each for each evaluation is:

(4) $$r=z\sqrt{\frac{metric(1-metric)}{N}}$$

*z* is the level of significance when *N* is the number test of samples. The values were calculated over the total confusion matrix, which contains the test fold outcomes from each experiment’s 5-fold cross-validation. The performance of hand detection using semantic segmentation networks was evaluated using Accuracy, Intersection over Union (IoU), and Dice Similarity Coefficient (DSC) metrics:

(5) $$WeightedAccuracy=\frac{TP+TN}{TP+TN+FP+FN}$$

(6) $$IoU=\frac{TP}{TP+FP+FN}$$

(7) $$DSC=\frac{2TP}{2TP+FP+FN}$$

The intersection over union (IoU) metric, also known as the Jaccard index, is a technique for quantifying the percentage of overlap between the ground true mask and the predicted mask. The main difference between DSC and IoU is that DSC counts double weight for TP pixels compared to IoU.

Here, TP = number of true positive instances, TN = number of true negative instances, FP = number of false-positive instances, and FN = number of false-negative instances.

The performance of sign recognition using ResNet18, MobileNet_V2 and EfficientNet_B1 was evaluated by Weighted Accuracy, Overall Accuracy, Precision, Sensitivity, F1_score, and Specificity:

(8) $$Precision=\frac{TP}{TP+FP}$$

Here, precision is the correctly classified positive sign classes among all the test images classified as the positive class for that sign class:

(9) $$Sensitivity=\frac{TP}{TP+FN}$$

The rate of correctly predicted test images in the positive class images is known as Sensitivity:

(10) $$Specificity=\frac{TN}{TN+FP}$$

Specificity is the measurement of the rate of accurately predicted negatives in the negatively identified samples:

(11) $$F1\_Score=\frac{2\times (Precision\times Sensitivity)}{Precision+Sensitivity}$$

where the harmonic mean of precision and sensitivity is known as F1_score:

(12) $$OverallAccuracy=\frac{TP}{TP+TN+FP+FN}$$

At last, the Overall accuracy is the rate of positive class among all the true positive, true negative, false positive, and false negative combined.

A receiver operating characteristic curve (ROC curve), which is a graph that depicts a classification model’s performance across every classification thresholds by plotting two parameters: (1) Recall/ True positive rate and (2) False positive rate are drawn for three modes for before and after background removal. The area under the curve (AUC) is calculated, which is the two-dimensional area underneath a ROC curve in the range from 0 to 1. The higher value of AUC demonstrates the ability of a model in distinguishing the true positive and negative classes:

(13) $$FalsePositiveRate=\frac{FN}{TP+FN}$$

### 3.8. Real-Time Bangla Sign Alphabets and Numerals Video Classification and Interpretation Technique

Videos are the consequent frames of images, and therefore there is a practice in the deep learning sector to consider real-time video classification to be equivalent of doing image classification *N* time if the number of frames in the video is *N*. However, in this case, the challenge appears as prediction flickering because classifying every frame can be miss-classified or the confidence level may be less than that desired. In this research, “Rolling Prediction Average” is adopted for real-time Bangla Sign Alphabets and Numerals interpretation. Algorithm 1 represents the algorithm of rolling average prediction in real-time Bangla Sign Alphabets and Numerals video classification interpretation.
**Algorithm 1** Real-time Bangla Sign Alphabets and Numerals video classification and interpretation by rolling prediction average**Input:** Real-time Bangla Sign Alphabets and Numerals video **Output:** real-time Bangla Sign Alphabets and Numerals video and corresponding label
1:**for**i←1 to *N* **do**2:    Pass each frame through the Bangla Sign Alphabets and Numerals recognition model;3:    Obtain predictions [$P_{1},P_{2}\cdots \cdots P_{n}$];4:    Make a list of last k prediction [$P_{1},P_{2},\cdots P_{k}$] for $P_{average}=\frac{1}{k}\sum_{m=1}^{k}P_{m}$;5:    Select label with the greatest probability;6:    Label the frame based on the greatest probability, write the output to disk and display the output image;7:    i+ = 1;8:**end for**9:Release the frame;

## 4. Results

The results for both classification with and without background approach are described in this section. The comparative analysis between the two approaches and the comparative analysis between previous findings with the best performed models in sign recognition is also reported in this section.

### 4.1. Classification with Background Approach

**Figure 6:**
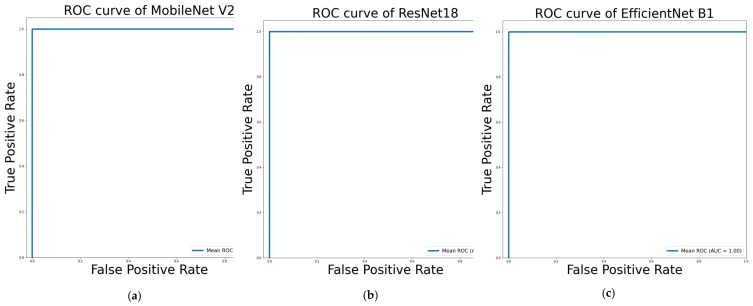
ROC curves of (**a**) MobileNet_V2, (**b**) ResNet18, (**c**) EfficientNet_B1 in classification with the background of Bangla Sign Language.

The performance of sign recognition using transfer learning on ResNet18, MobileNet_V2, and EfficientNet_B1 is tabulated in Table 3. ResNet18 surpassed MobileNet_V2 and EfficientNet_B1 in terms of overall accuracy, precision, sensitivity, F1 score, and specificity after five-fold cross-validation. The highest overall accuracy was achieved 99.99% using ResNet18, while the least 99.05% overall accuracy was achieved using EfficientNet_B1. ResNet18 had the highest trainable parameters which is more than 11M, MobileNet_V2 has only 0.08% less accuracy with having almost 5 times less the number of trainable parameters. Specificity or the proportion of negative class sample identification to the negatively class samples by ResNet18, and MobileNet_V2 according to Equation (10) was found to be the same as 100%. From Equations (8)–(11), it is perceptible that the instances of False positive and False negative recognition of signs are the highest by EfficientNet_B1 because it performed with the lowest precision, sensitivity, and F1 score of 99.07%, 99.05%, and 99.06% respectively. All three of the CNN networks performed above 99% overall accuracy, which reflects that the pre-trained networks can perform well for the sign recognition of such a large class (87 classes) image domain problem even in the presence of a wide range of background changes. Inference time (seconds) is an indication of models taking time to classify one image properly. EfficientNet_B1 took the highest time 0.0253, while MobileNet_V2 was the fastest with an inference time of 0.0091 s. Figure 6 illustrates the ROC curves of MobileNet_V2, ResNet18, and EfficientNet_B1. The Loss, accuracy curves can be found in Tables S1–S3, and better resolution ROC curves can be found in Figures S1–S3 for EfficientNet_B1, MobileNet_V2, and ResNet18 respectively.

### 4.2. Classification without Background Approach

The performance of the classification without background approach can be evaluated by the performance of two units, (1) Hand Detection and (2) Sign Recognition.

#### 4.2.1. Hand Detection

**Figure 7:**
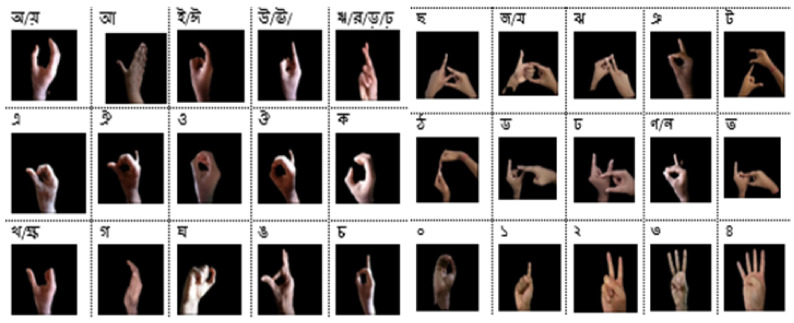
Representation of Segmented BdSL-D1500 dataset using DenseNet201 FPN.

The performance of the hand detection using M-UNet, DenseNet 201 FPN, and UNet is tabulated in Table 4. Different loss function was applied in these segmentation networks to find the best model by comparative analysis on loss, accuracy, IoU, and DSC of the five-fold cross-validation results. DenseNet 201 FPN with Dice loss outperformed the other combination of segmentation networks and loss functions. All three segmentation networks showed more than 98% accuracy, while DenseNet201 FPN performed the highest accuracy of 98.644%. DenseNet201 FPN with Dice Loss achieved the highest IoU and DSC 93.448% and 96.524%, respectively, which indicates that the model is capable of detecting most of the regions of the hand reliably. M-UNet with Dice loss detected less or more area overlapped with ground truth of a hand region. Thus, this model performed the lowest in IoU and DSC, which indicates that the false positive and false negative detection is highest in this model. Figure 7 is a representation of segmented BdSL-D1500 dataset using DenseNet 201 FPN.

#### 4.2.2. Sign Recognition

Using the best model found in hand detection, which is DenseNet201-FPN, the backgrounds from images of the BdSLD-1500 dataset were removed. The performance evaluation of five-fold cross-validation of ResNet18, MobileNet_V2, and EfficientNet_B1 as a Sign recognition model on this hand-detected dataset is carried out.

**Figure 8:**
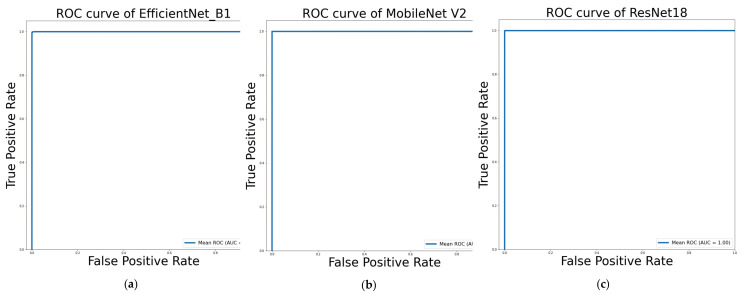
ROC curves of (**a**) EfficientNet_B1; (**b**) MobileNet_V2; (**c**) ResNet18 in classification without the background of Bangla Sign Language.

The performance of sign recognition models from background removed images is tabulated in Table 5. In this approach, MobileNet_V2 outperformed the ResNet18 and EfficientNet_B1, while ResNet18 and EfficientNet_B1 had more trainable parameters. The overall accuracy, precision, sensitivity, and F1_score of the ResNet18 and MobileNet_V2 were over 99%. The models ResNet18, MobileNet V2, and EfficientNet B1 exhibit 100% specificity, 100% specificity, and 99.98% specificity, respectively, showing that they have an extremely low false alarm rate. Despite having more parameters than MobileNet V2, EfficientNet B1 had the lowest performance of the three CNNs used in this sign recognition problem. However, the overall accuracy precision, sensitivity, and F1 score are over 98% for EfficientNet, which indicates that the model is not the best performer for sign recognition even though this is the deepest network among the three networks. The lowest inference time was found for MobileNet_V2 with 0.0092 seconds while EfficientNet_B1 took the highest 0.0244 second inference time. Figure 8 illustrates the ROC curves of EfficientNet_B1, MobileNet_V2, and ResNet18 in Bangla Sign Language Recognition without background approach.

### 4.3. Comparative Analysis between the Classification with Background and Classification without Background Approaches

In the classification with background approach, ResNet18, MobileNet_V2, and EfficientNet_B1 achieved 100% accuracy for 74, 76, and 20 classes of signs, respectively. In the classification without background approach, ResNet18, MobileNet_V2, and EfficientNet_B1 achieved 100% accuracy for 72,78, and 17 classes of signs, respectively. The lowest accuracy among three CNN models in the first approach achieved 99.85% by EfficientNet_B1 to recognize এ, while the same CNN architecture achieved the lowest 99.80% accuracy recognizing ঊ in the second approach. Comparing Table 3 and Table 5, it is also evident that ResNet18 in the first approach performed the best by evaluating overall accuracy, precision, sensitivity, F1 score, and specificity results. The slightly low performance of the second approach compared to the first (classification with background) can be understood in this way—that any CNN model can perform better if it gets more information in the images to learn; however, it is important to see whether the network is learning from the hand area of the images or it is learning from the backgrounds to differentiate the classes. In both cases, the overall accuracy is more than 99%, which indicates that both approaches can be feasible for implementation for sign recognition and interpretation; however, this can be confirmed from the image visualization results which are reported in the next section. The Loss, accuracy curves can be found in Tables S4–S6, and better resolution ROC curves can be found in Figures S4–S6 for EfficientNet_B1, MobileNet_V2, and ResNet18 respectively.

### 4.4. Visualization Using CAM, Smoothed Grad-CAM++, and Score-CAM

Table 6 represents the comparative recognition and localization analysis of Bangla Sign Alphabets and Numerals using classification with and without backgrounds. In this work, three visualization techniques (CAM, Smoothing Grad-CAM++, and Score-CAM) were used to help better grasp the BdS Alphabets and Numerals recognition for different CNN models for two classification schemes. In the first approach, the hand region is detected as the region of interest for recognition, which can be understood in such a way that the model is predicting Bangla Sign Alphabets and Numerals based on the hand features. As hand segmented image is used in the training of second approach, it is also found that MobileNet_V2 learned more from the hand region rather than the black background for sign alphabets and numerals recognition. This visualization of both approaches shows that, for this problem, CNN is not making a decision from non-relevant regions as reported by the fact that CNN makes a decision on a non-relevant region of the image and is thus unreliable. In Table 6, Bangla Sign Numerals and Bangla Sign Alphabets (one-hand and two-hand representation) were visualized using CAM, Smoothed Grad-CAM, and Score-CAM for better understanding and bringing reliability on CNN about predicting Bangla Sign Alphabets and numerals.

### 4.5. Related Works and Performance Analysis

Table 7 compares the performance of different approaches that have been published in the literature for Bangla Sign Alphabets and Numerals recognition with our proposed methods. The dataset used in this research contains the highest number of signs and incorporated both one-hand and two-representation in the same model, which was unique compared to others. The dataset also contains the highest number of images used for Bangla Sign Alphabets and Numerals recognition so far. It is evident from the table that ResNet18 for classification with background outperformed the other techniques. The classification without background approach adopted in this research also performed better than other techniques but [19]. Overall, both of the approaches in this research produced outstanding accuracy in Bangla Sign Alphabets and Numerals recognition.

### 4.6. Real-Time Bangla Sign Alphabets and Numerals Video Classification and Interpretation

**Figure 9:**
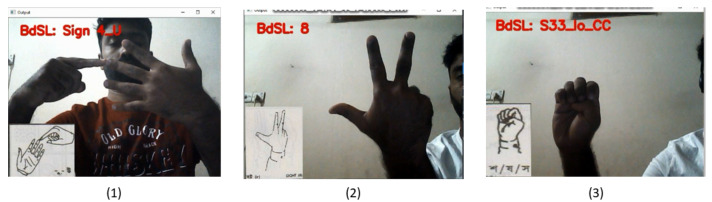
Real-time Bangla Sign Alphabets and Numeral interpretation (1) উ (left, class name: Sign 4_U), (2) ৮ (middle, class name: 8), (3) শ/ষ/স (right, class name: S33_lo_CC).

The real-time Bangla Sign Alphabets and Numerals interpretation were done using videos as input captured by a webcam. The prediction flickering was eliminated using rolling average prediction. The number of k=10 prediction window was taken to make a list for average prediction and choosing the label based on the corresponding highest probability. Figure 9 demonstrates real-time interpretation of two different representations (one-handed and two-handed) of Bangla Sign Alphabets and one Numeral interpretation. In real-time sign video classification and interpretation, the ResNet18 model trained for classification with background approach was used because this model performed best among all other models in two approaches.

## 5. Conclusions

A real-time Bangla Sign Language interpreter can enable more and more people to the mainstream workforce in Bangladesh. With the Bangla Sign Alphabets and Numerals interpreter, both one-handed and two-handed representations of Bangla Sign Alphabets were enabled. It was tried to compare the classification with background approach and classification without background approaches to determine the best working model for BdS Alphabets and Numerals interpretation, and the CNN model trained with the images that had background was found to be more effective than without background. The hand detection portion in the segmentation approach must be more accurate in the hand detection process to boost the overall accuracy in the sign recognition. With different visualization technique and performance metrics, it was found that ResNet18 in the first approach performed best with 99.99% accuracy, precision, F1 score, sensitivity, and 100% specificity. In this study, the model’s accuracy was found to be much higher than previous literature when BdS Alphabets and Numerals recognition is compared. This dataset which is being provided in this study comprises the biggest accessible dataset for BdS Alphabets and Numerals in order to reduce inter-class similarity while dealing with diverse image data, which comprises various backgrounds and skin tones. This dataset is publicly available for researchers to support and encourage further research on Bangla Sign Language Interpretation so that the hearing and speech-impaired individuals can benefit from this research.

## 6. Recommendations

An accurate and efficient real-time Bangla Sign Language interpreter has versatile implementation in the education sector, daily life, medical sector, etc. This research is based on the alphabets and numerals interpretation, but to establish a user friendly and effective system for sign language interpretation, sign words, and sentences must be incorporated for meaningful conversion between a sign language user and non-sign language user. Vision Transformers [45,46,47] are gaining attention and slowly replacing CNNs in so many tasks. Vision transformers can be implemented as future investigation for Bangla Sign Language interpretation systems. In the future, the research will expand to this area to incorporate the sign words and sentences. Domain adaptation [48] will be also a future goal as real-time applications include the population which belongs to the different distributions than the training and validation data. In addition, the real-time application is done using the webcam as an input device but to make it more user oriented a smart phone implementation of this research will be a future goal.

## Figures and Tables

**Table 1:** Mean and Standard Deviation values of BdSL-D1500 and BdSLHD-2300 dataset.

Dataset	Mean			Standard Deviation		
	R	G	B	R	G	B
BdSl-D1500	0.4469	0.4164	0.4054	0.2081	0.2193	0.2232
BdSLHD-2300	0.4594	0.4273	0.4145	0.2448	0.2555	0.2713

**Table 2:** Training parameters used in a classification without background approach.

Training Parameters	Hand Detection	Sign Recognition
Batch Size	16	16
number of Folds	5	5
Learning Rate	0.001	0.0001
Learning Rate Drop Factor	0.1	0.1
Max Epoch	50	10
Epochs Patience	3	3
Epochs Stopping Criteria	6	3
Loss Function	NLLLoss	NLLLoss
	DiceLoss	
	BCELoss	

**Table 3:** Different performance matrices of CNN Models in Classification with a background approach for BdS Alphabets and Numerals recognition.

Model	Parameters	Inference Time (s)	Overall Accuracy	Precision	Sensitivity	F1 Score	Specificity	AUC
ResNet18	11,221,143	0.0129	99.99	99.99	99.99	99.89	100.00	1.00
MobileNet_V2	2,335,319	0.0091	99.91	99.91	99.91	99.91	100.00	1.00
EfficientNet_B1	6,624,631	**0.0253**	99.05	99.07	99.05	99.06	99.99	1.00

**Table 4:** Different performance matrices of hand detection models.

Model	Loss Function	Loss	Accuracy	IoU	DSC
M-UNet	NLL	0.044	98.438	92.554	95.992
	BCE	0.044	98.490	92.778	96.130
	DICE	0.044	98.278	91.852	95.576
DenseNet201 FPN	NLL	0.036	98.584	93.104	96.342
	BCE	0.037	98.580	93.050	96.308
	**DICE**	**0.035**	**98.644**	**93.448**	**96.524**
UNet	NLL	0.044	98.382	92.282	95.846
	BCE	0.044	98.442	92.556	96.004
	DICE	0.042	98.344	92.194	95.782

**Table 5:** Different performance matrices of classification without background models for BdS Alphabets and Numerals recognition.

Model	Parameters	Inference Time (s)	Overall Accuracy	Precision	Sensitivity	F1 Score	Specificity	AUC
ResNet18	11,221,143	0.0127	99.88	99.88	99.88	99.88	100.00	1.00
MobileNet_V2	2,335,319	0.0092	99.91	99.91	99.91	99.91	100.00	1.00
EfficientNet_B1	6,624,631	0.0244	98.61	98.65	98.61	98.60	99.98	1.00

**Table 6:** Visualization of Sign Language Recognition by ResNet18 and MobileNet_V2 by the classification with background approach and the classification without background approach, respectively.

Approach	BdSL Alphabet	Visualization			
		Input Image	CAM	Smoothed Grad-CAM++	Score-CAM
Classification with background approach	২	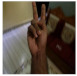	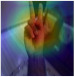	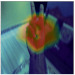	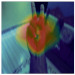
	ঐ	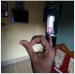	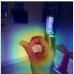	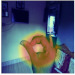	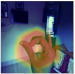
	ই	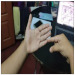	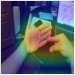	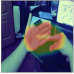	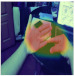
Classification without background approach	২	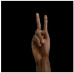	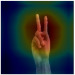	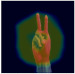	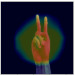
	ঐ	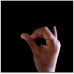	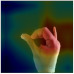	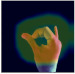	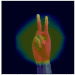
	ই	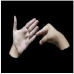	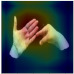	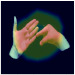	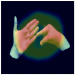

**Table 7:** Comparative analysis of the performance of different sign language recognition models reported in the literature and our proposed model.

Reference	Technique Used	Dataset			Recognition Accuracy (%)
		Sign	Training	Testing	
[10]	Image processing, SVM	15	240	570	86.00
[42]	Haar Cascade Classifier	36	3600	7200	88.89
[17]	VGG19	38	11,061	1520	89.60
[43]	CNN-LSTM	36	10,800	300	88.50
[12]	Window Grid Vector	52	5200	5200	95.50
[44]	CNN	45	27,824	3091	99.80
[19]	Color-coded Fingertip, ResNet18	37	36,766	9192	99.97
Our proposed method	Classification with background approach ResNet18	87	105,648	26,412	**99.99**
	Classification without background approach DenseNet201 FPN - MobileNet_V2				99.91

## Data Availability

Datasets are publicly available in [31,34]. All the results can be found in shorturl.at/grDLV.

## Supplementary Materials

The following are available at https://www.mdpi.com/article/10.3390/s22020574/s1, Table S1: Accuracy and Loss curves of EfficientNet B1 training in the “Classification with Backgrounds” Approach, Table S2: Accuracy and Loss curves of MobileNet V2 training in the “Classification with Backgrounds” Approach, Table S3: Accuracy and Loss curves of ResNet18 training in the “Classification with Backgrounds” Approach, Table S4: Accuracy and Loss curves of EfficientNet B1 training in the “Classification without Backgrounds” Approach, Table S5: Accuracy and Loss curves of MobileNet V2 training in the “Classification without Backgrounds” Approach, Table S6: Accuracy and Loss curves of ResNet18 training in the “Classification without Backgrounds” Approach, Figure S1: ROC curve of EfficientNet B1 in the “Classification with Background” approach, Figure S2: ROC curve of MobileNet V2 in the “Classification with Background” approach, Figure S3: ROC curve of Resnet18 in the “Classification with Background” approach, Figure S4: ROC curve of EfficientNet B1 in the “Classification without Background” approach, Figure S5: ROC curve of MobileNet V2 in the “Classification without Background” approach, Figure S6: ROC curve of Resnet18 in the “Classification without Background” approach. A supporting video article is available at: KKP/BdSL_recognition_system.
