# Supplementary material for: Bangla Sign Language (BdSL) Alphabets and Numerals Classification Using a Deep Learning Model
Source: Sensors (Basel). 2022 Jan 12;22(2):574. doi: 10.3390/s22020574 (PMC8780505; doi:10.3390/s22020574)
Supplement: Supplementary file 1 [file sensors-22-00574-s001.zip › sensors-1467493-supplementary.pdf]

sensors

Supplementary Materials

Article

Bangla Sign Language (BdSL) Alphabets and Numerals Classification Using a Deep Learning Model

Kanchon Kanti Podder [1], Muhammad E. H. Chowdhury [2,*], Anas M. Tahir [2], Zaid Bin Mahbub [3], Amith Khandakar [2], Md Shafayet Hossain [4] and Muhammad Abdul Kadir [1]

[1] Department of Biomedical Physics & Technology, University of Dhaka, Dhaka 1000, Bangladesh; kanchon.k.podder@bmpt.du.ac.bd (K.K.P.); kadir@du.ac.bd (M.A.K.)
[2] Department of Electrical Engineering, Qatar University, Doha 2713, Qatar; a.tahir@qu.edu.qa (A.M.T.); amitk@qu.edu.qa (A.K.)
[3] Department of Mathematics and Physics, North South University, Dhaka 1229, Bangladesh; zaid.mahbub@northsouth.edu
[4] Department of Electrical, Electronic and Systems Engineering, Universiti Kebangsaan Malaysia, Bangi 43600, Selangor, Malaysia; p108100@siswa.ukm.edu.my
* Correspondence: mchowdhury@qu.edu.qa

Citation: Podder, K.K.; Chowdhury, M.E.H.; Tahir, A.M.; Mahbub, Z.B.; Khandakar, A.; Hossain, M.S.; Kadir, M.A. Bangla Sign Language (BdSL) Alphabets and Numerals Classification Using a Deep Learning Model. *Sensors* **2022**, *22*, 574. https://doi.org/10.3390/s22020574

Academic Editor: Petros Daras

Received: 1 November 2021
Accepted: 23 December 2021
Published: 12 January 2022

Publisher's Note: MDPI stays neutral with regard to jurisdictional claims in published maps and institutional affiliations.

Copyright: © 2022 by the authors. Submitted for possible open access publication under the terms and conditions of the Creative Commons Attribution (CC BY) license (https://creativecommons.org/licenses/by/4.0/).

Model: EfficientNet B1

The accuracy and loss curves during the training of EfficientNet B1 is given Table S1:

Table S1. Accuracy and Loss curves of EfficientNet B1 training in the "Classification with Backgrounds" Approach.

Fold	Accuracy	Loss
	Classifications with Backgrounds	
1	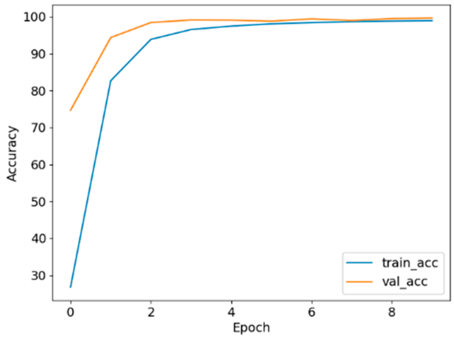	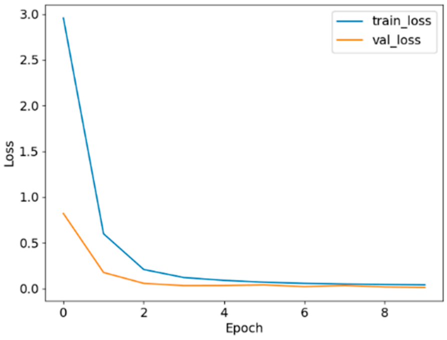
2	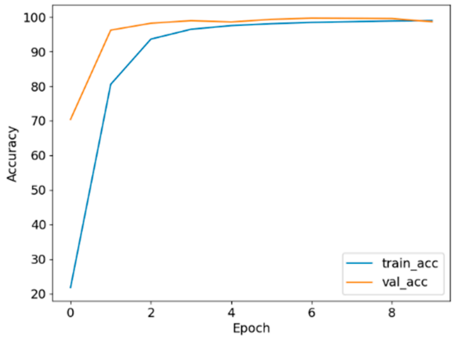	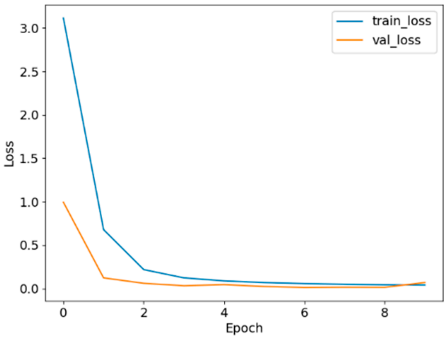
3	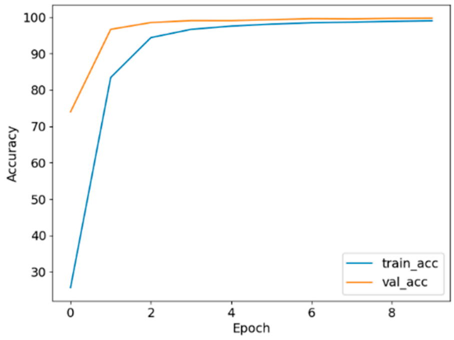	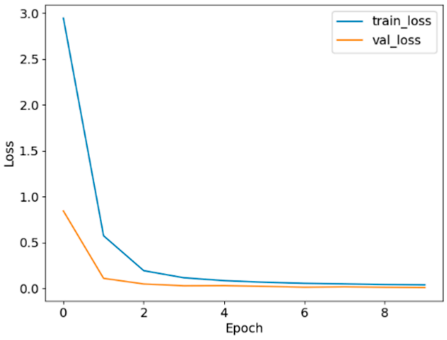
	Classifications with Backgrounds	
4	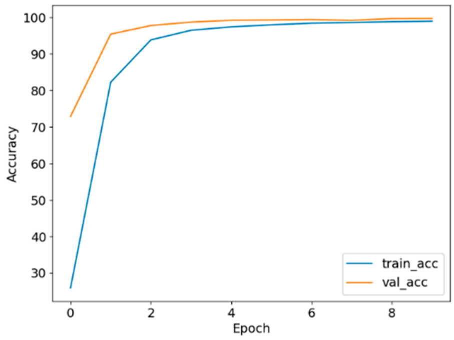	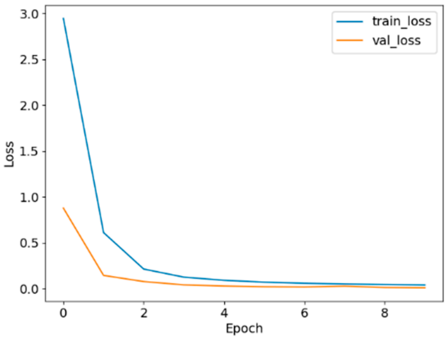

5	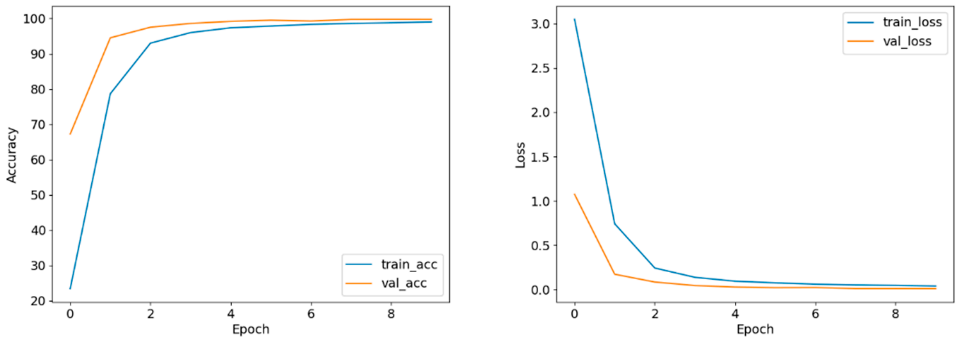

ROC Curve:

The ROC Curve of EfficientNet B1 in the “Classification with Background” is given below:

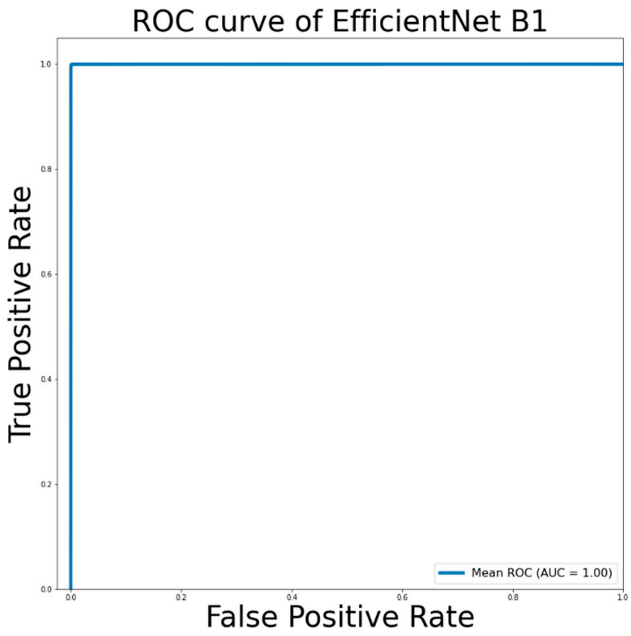

Figure S1. ROC curve of EfficientNet B1 in the "Classification with Background" approach.

Model: MobileNet V2

The accuracy and loss curves during the training of MobileNet V2 is given Table S2:

Table S2. Accuracy and Loss curves of MobileNet V2 training in the "Classification with Backgrounds" Approach.

Fold	Accuracy	Loss
	Classifications with Backgrounds	
1	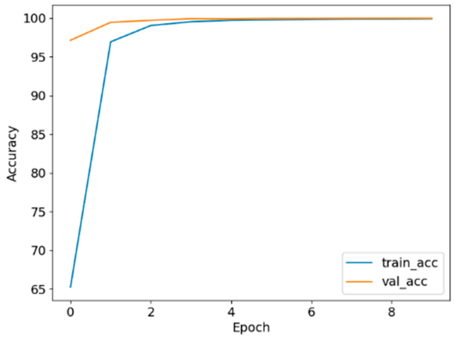	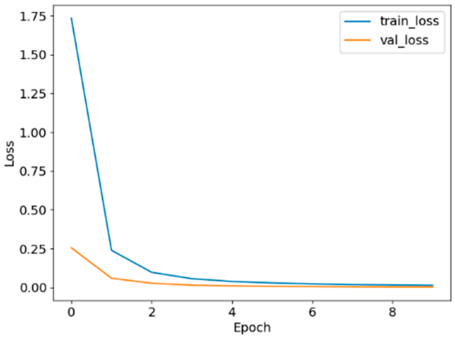
2	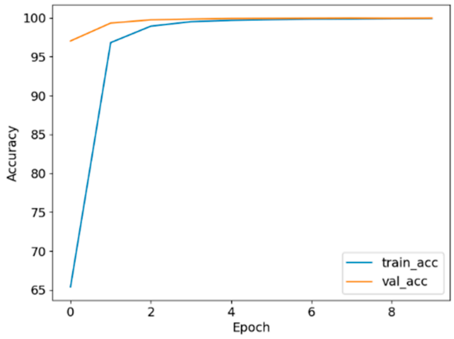	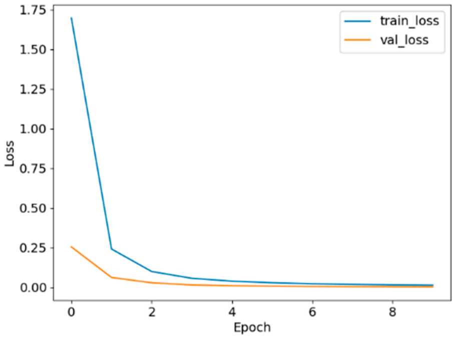
3	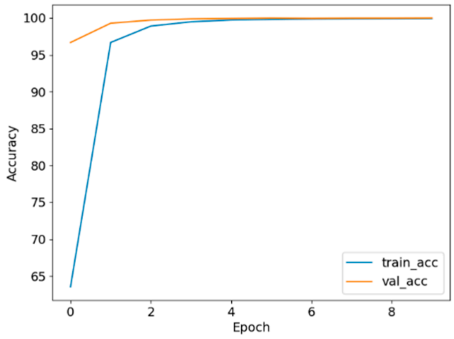	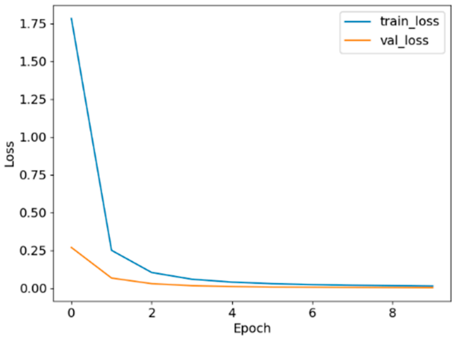
	Classifications with Backgrounds	
4	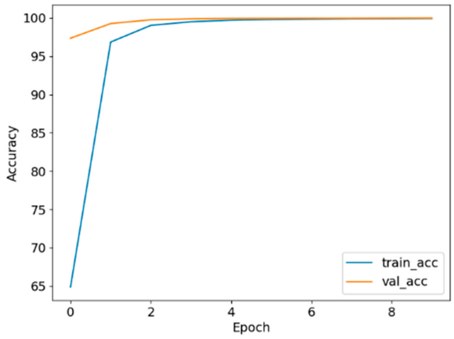	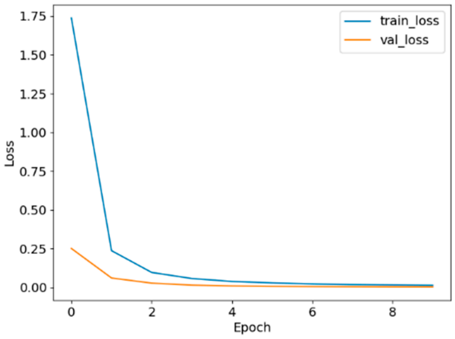

5	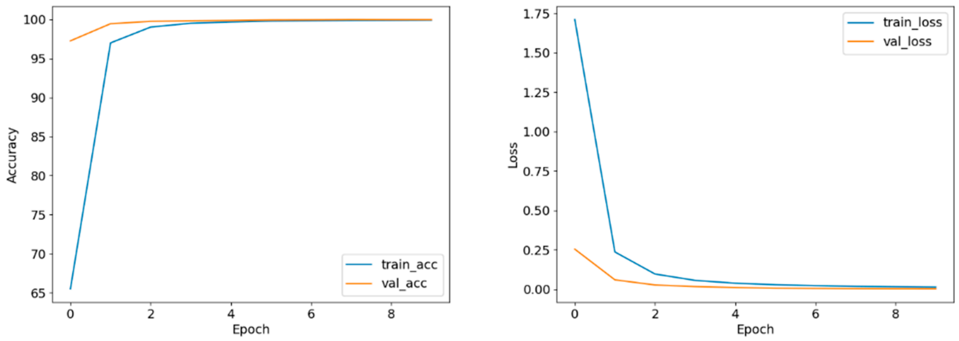

ROC Curve:

The ROC Curve of MobileNet V2 in “Classification with Background” is given below:

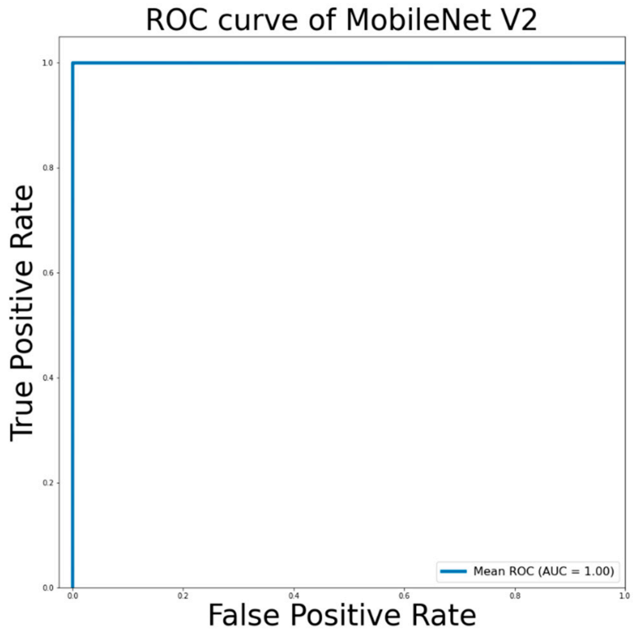

Figure S2. ROC curve of MobileNet V2 in the "Classification with Background" approach.

Model: ResNet18

The accuracy and loss curves during the training of ResNet18 is given Table S3:

Table S3. Accuracy and Loss curves of ResNet18 training in the "Classification with Backgrounds" Approach.

Fold	Accuracy	Loss
	Classifications with Backgrounds	
1	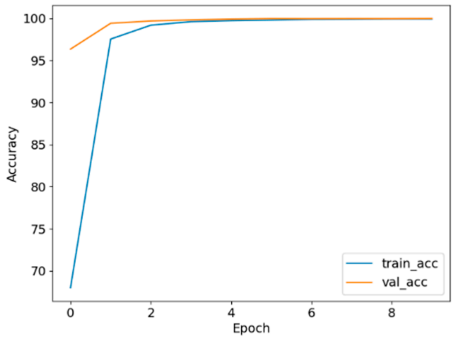	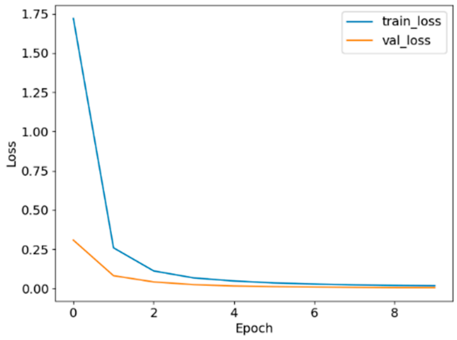
2	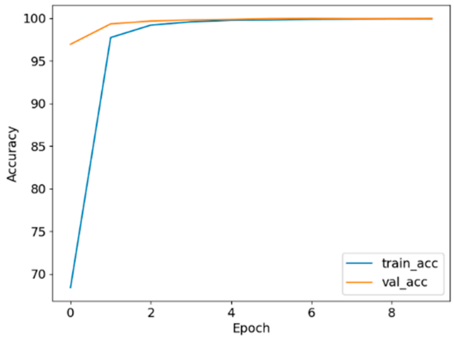	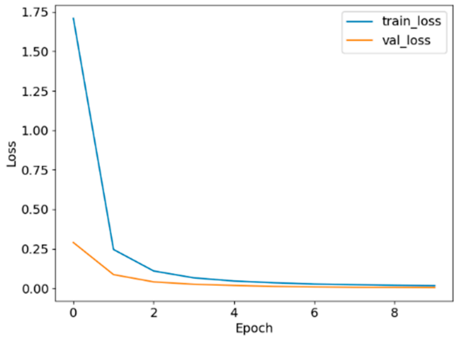
3	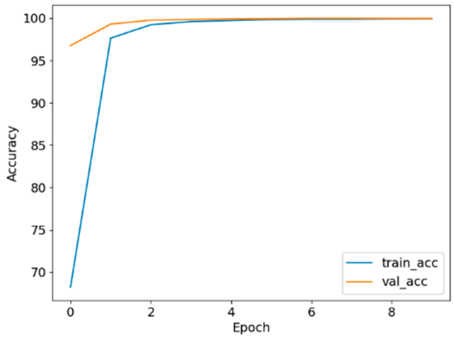	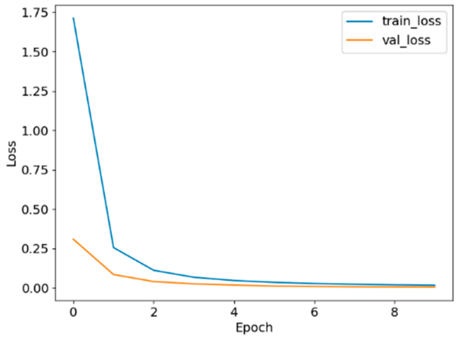
	Classifications with Backgrounds	
4	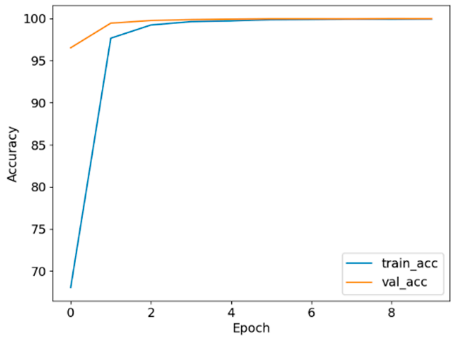	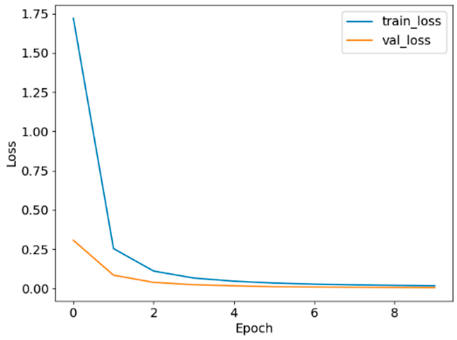

5	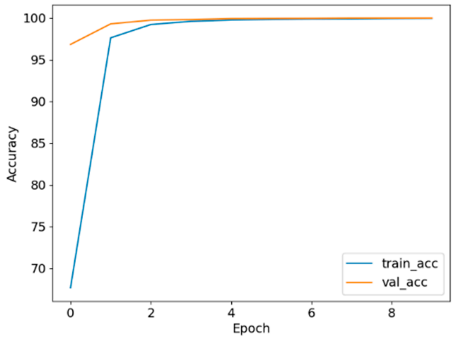	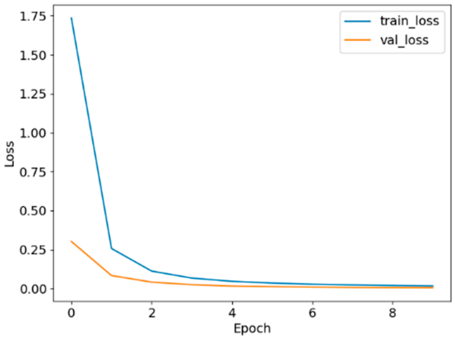

ROC Curve:

The ROC Curve of ResNet18 in "Classification with Background" is given below:

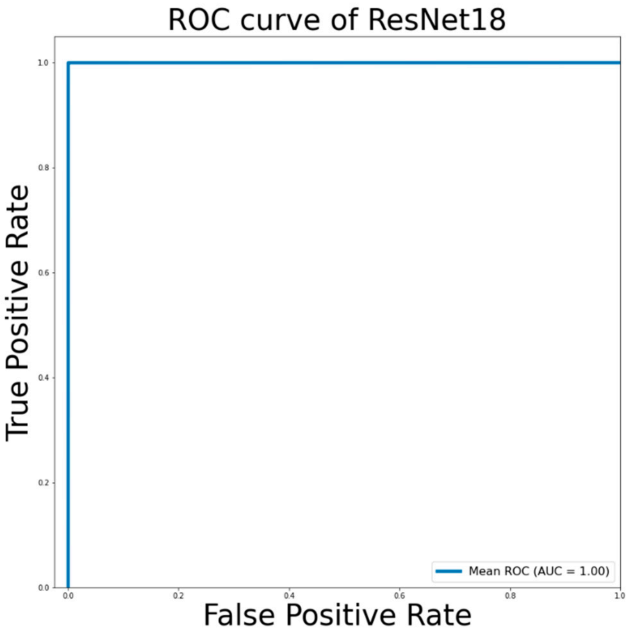

Figure S3. ROC curve of ResNet18 in the "Classification with Background" approach.

Model: EfficientNet B1

The accuracy and loss curves during the training of EfficientNet B1 is given Table S4:

Table S4. Accuracy and Loss curves of EfficientNet B1 training in the "Classification without Backgrounds" Approach.

Fold	Accuracy	Loss
	Classifications without Backgrounds	
1	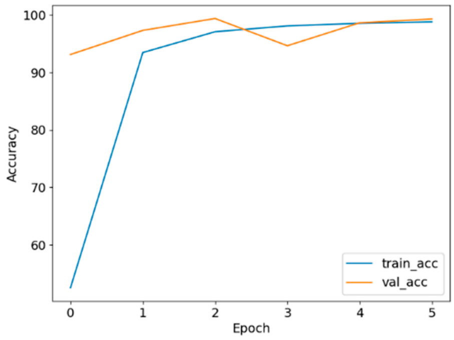	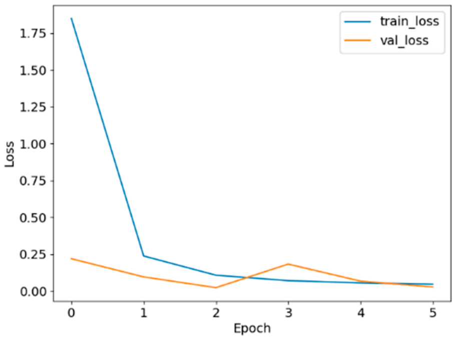
2	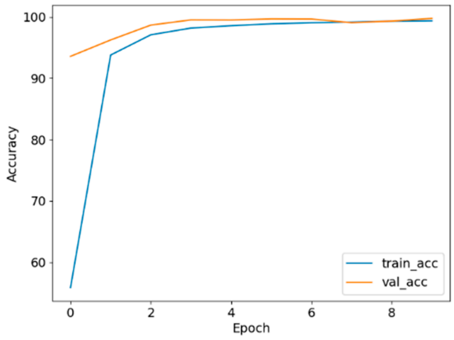	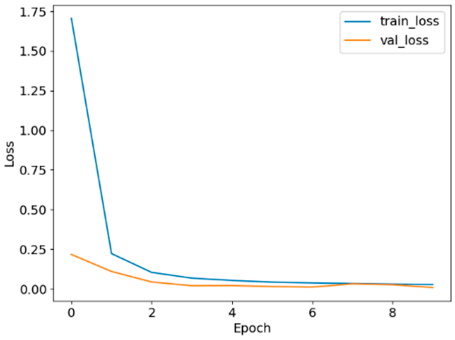
3	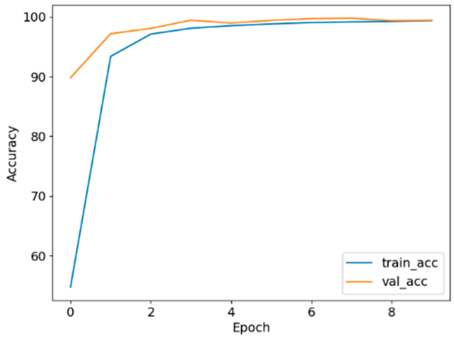	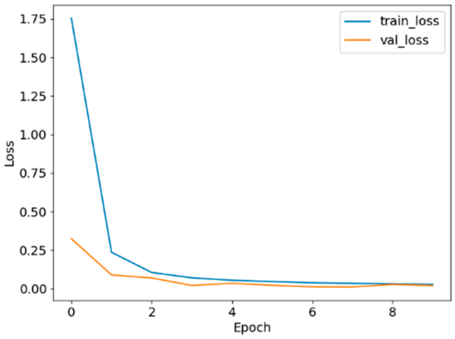
	Classifications without Backgrounds	
4	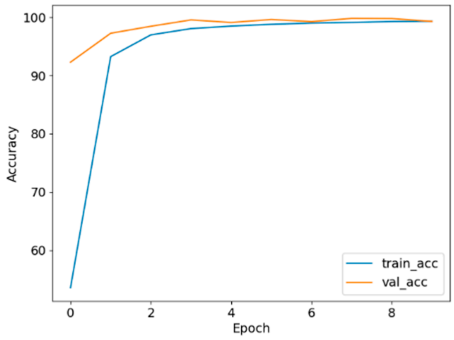	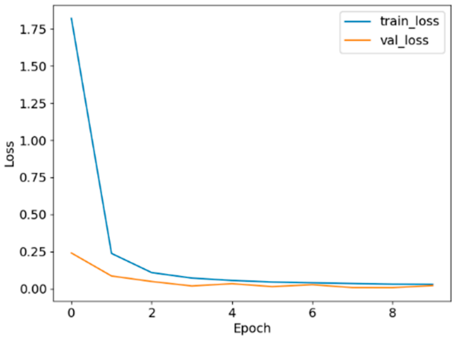

5	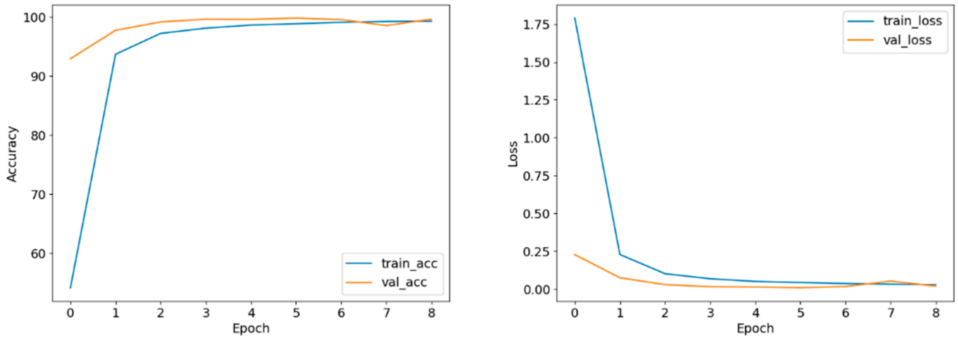

ROC Curve:

The ROC Curve of EfficientNet B1 in "Classification without Background" is given below:

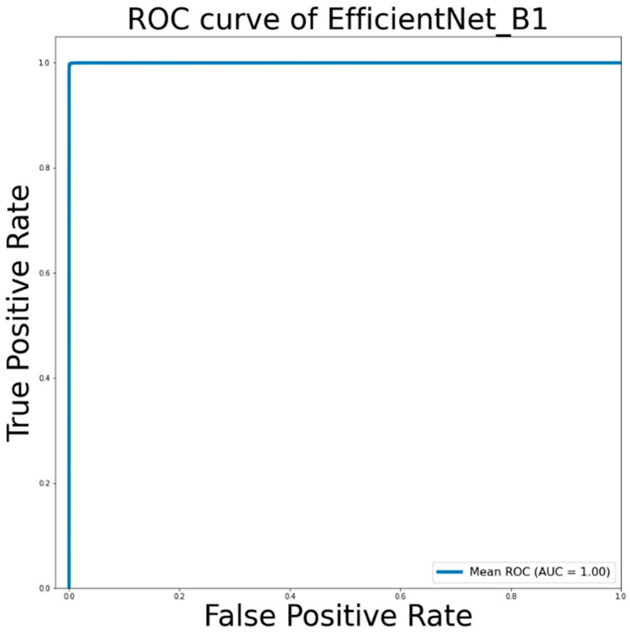

Figure S4. ROC curve of EfficientNet B1 in the "Classification without Background" approach.

Model: MobileNet V2

The accuracy and loss curves during the training of MobileNet V2 is given Table S5:

Table S5. Accuracy and Loss curves of MobileNet V2 training in "Classification Without Backgrounds" Approach.

Fold	Accuracy	Loss
	Classifications without Backgrounds	
1	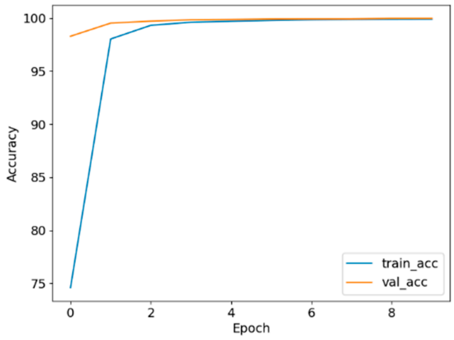	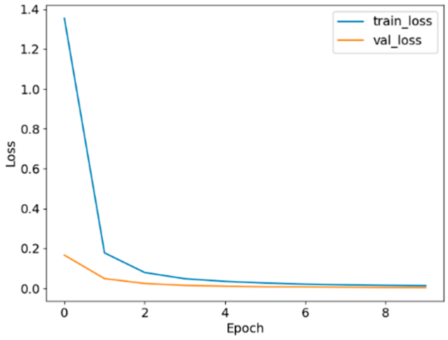
2	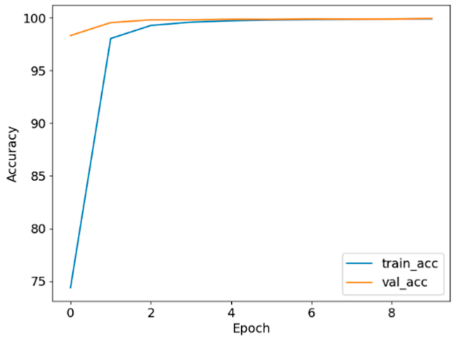	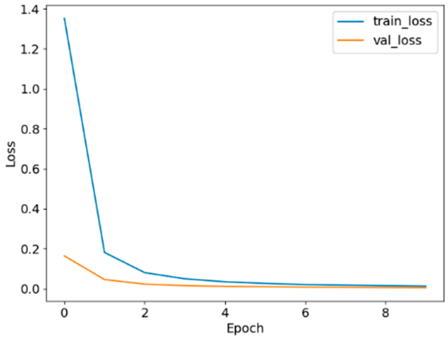
3	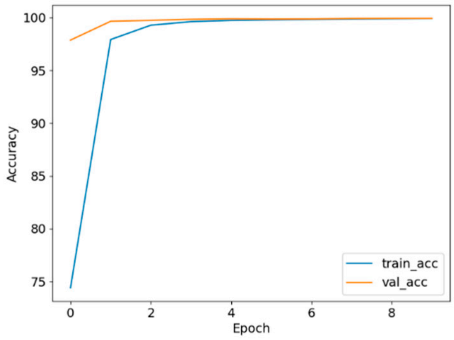	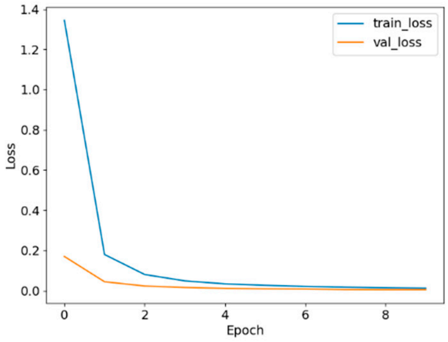
	Classifications without Backgrounds	
4	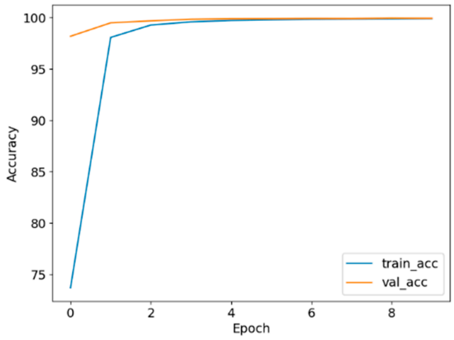	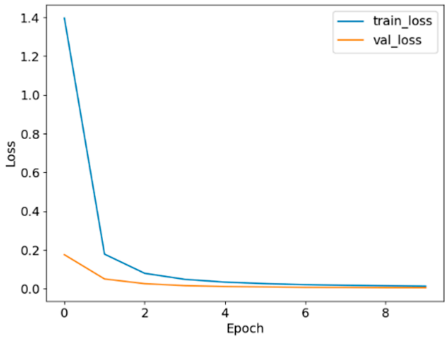

5	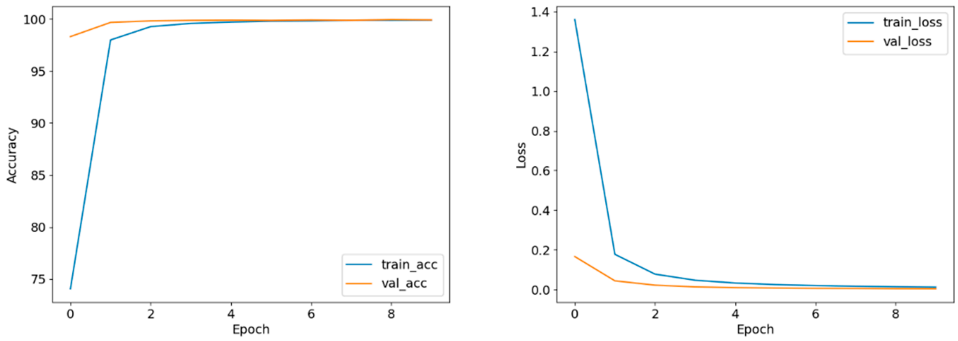

ROC Curve:

The ROC Curve of MobileNet V2 in "Classification without Background" is given below:

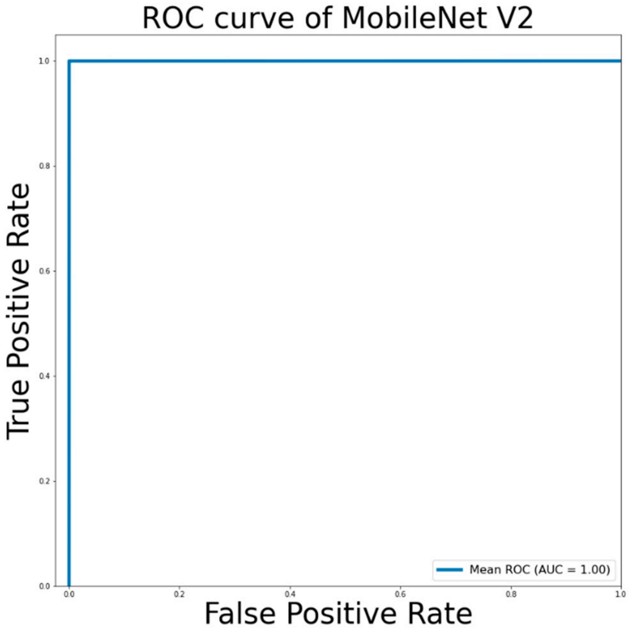

Figure S5. ROC curve of MobileNet v2 in "Classification without Background" approach.

Model: ResNet18

The accuracy and loss curves during the training of ResNet18 is given Table S6:

Table S6. Accuracy and Loss curves of ResNet18 training in "Classification Without Backgrounds" Approach.

Fold	Accuracy	Loss
	Classifications with Backgrounds	
1	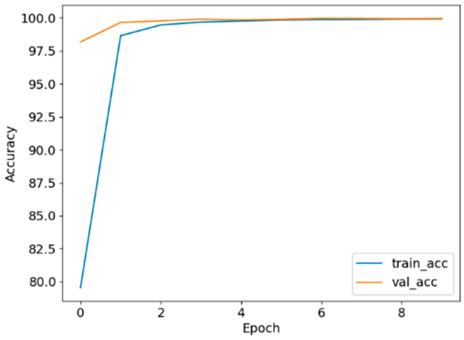	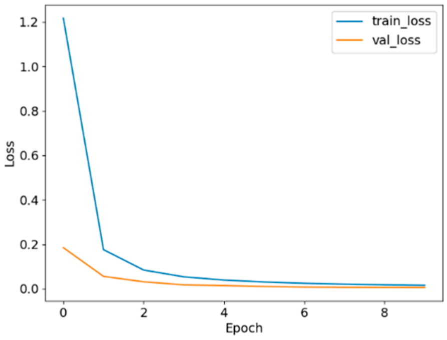
2	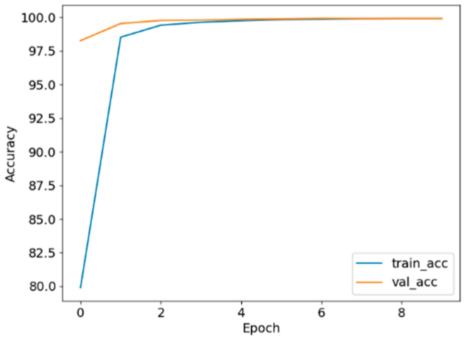	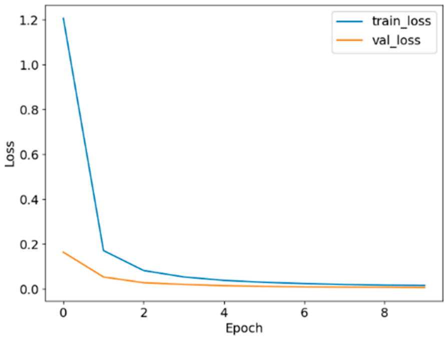
3	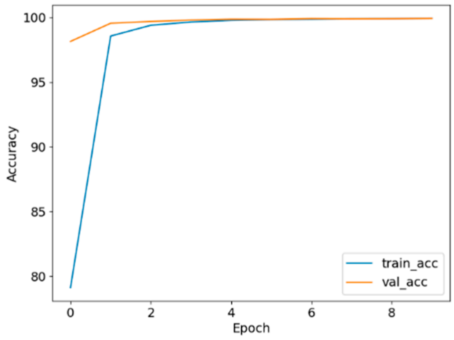	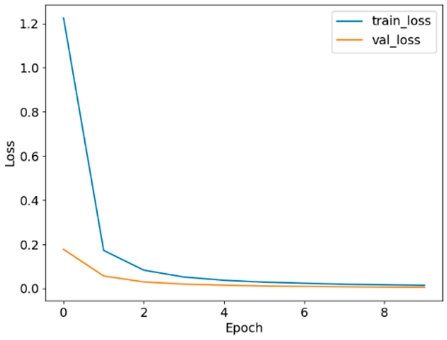
	Classifications without Backgrounds	
4	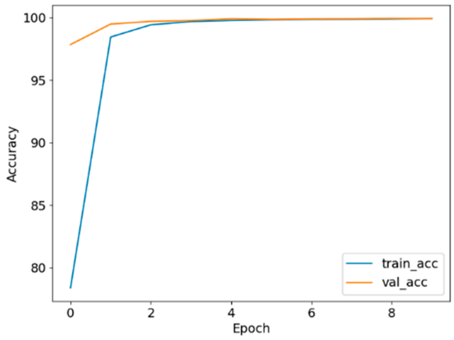	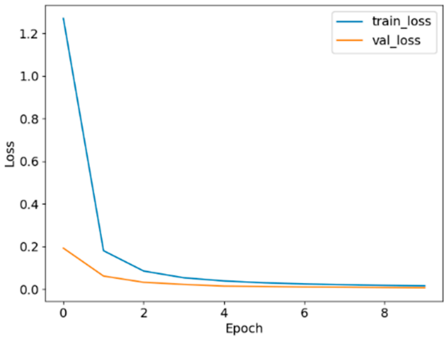

5	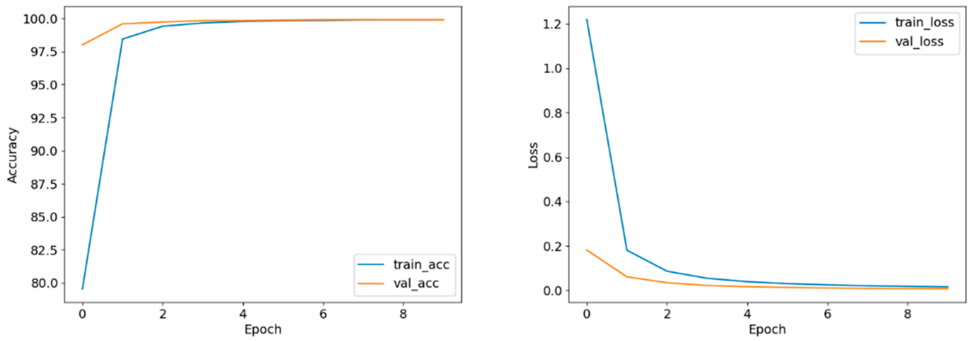

ROC Curve:

The ROC Curve of ResNet18 in “Classification without Background” is given below:

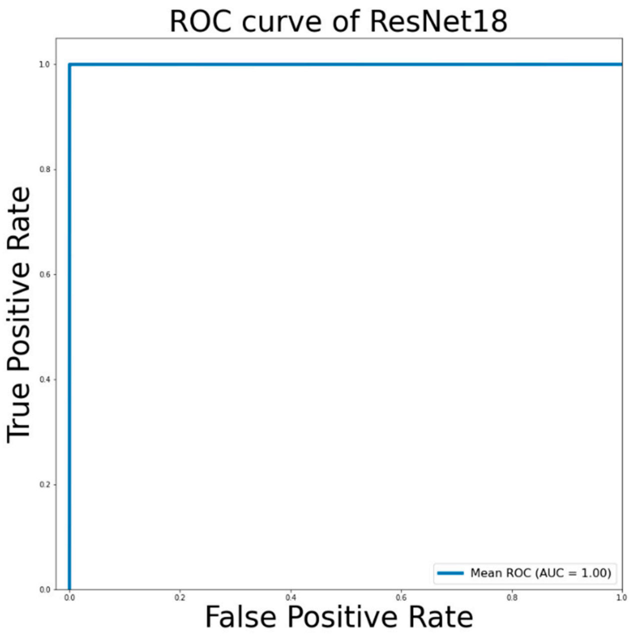

Figure S6. ROC curve of ResNet18 in "Classification without Background" approach.
